# Establishing a reference focal plane using convolutional neural networks and beads for brightfield imaging

**DOI:** 10.1038/s41598-024-57123-w

**Published:** 2024-04-02

**Authors:** Joe Chalfoun, Steven P. Lund, Chenyi Ling, Adele Peskin, Laura Pierce, Michael Halter, John Elliott, Sumona Sarkar

**Affiliations:** 1 National Institute of Standards and Technology, Boulder, CO USA; 2 National Institute of Standards and Technology, Gaithersburg, MD USA

**Keywords:** Optical microscopy, Image processing, Convolutional neural network, Reference focal plane, Cell biology, Cellular imaging, Computational science, Computer science, Information technology, Scientific data

## Abstract

Repeatability of measurements from image analytics is difficult, due to the heterogeneity and complexity of cell samples, exact microscope stage positioning, and slide thickness. We present a method to define and use a reference focal plane that provides repeatable measurements with very high accuracy, by relying on control beads as reference material and a convolutional neural network focused on the control bead images. Previously we defined a reference effective focal plane (REFP) based on the image gradient of bead edges and three specific bead image features. This paper both generalizes and improves on this previous work. First, we refine the definition of the REFP by fitting a cubic spline to describe the relationship between the distance from a bead’s center and pixel intensity and by sharing information across experiments, exposures, and fields of view. Second, we remove our reliance on image features that behave differently from one instrument to another. Instead, we apply a convolutional regression neural network (ResNet 18) trained on cropped bead images that is generalizable to multiple microscopes. Our ResNet 18 network predicts the location of the REFP with only a single inferenced image acquisition that can be taken across a wide range of focal planes and exposure times. We illustrate the different strategies and hyperparameter optimization of the ResNet 18 to achieve a high prediction accuracy with an uncertainty for every image tested coming within the microscope repeatability measure of 7.5 µm from the desired focal plane. We demonstrate the generalizability of this methodology by applying it to two different optical systems and show that this level of accuracy can be achieved using only 6 beads per image.

## Introduction

Automated workflows for image-based cell measurements require high-quality images consistently acquired at appropriate focal planes. Image-based cell assays are widely used to characterize cells, from basic research to the evaluation of cell-based products^1^. To ensure repeatable and reproducible measurements, it is important to control key image quality factors that can impart bias and variability^2,3^. Cell viability assays using trypan blue staining, for example, have been used for many decades to distinguish between live and dead cells^4^. Live cells possess intact membranes that do not allow many substances from their environment to pass through, such as dyes like trypan blue. Dead cells do not maintain impermeable membranes and will take up the trypan blue stain, giving the cells a dark blue appearance when they are observed. These assays are often automated or semi-automated to reduce user bias and improve measurement quality and throughput. Image focus is a critical parameter in this bright field imaging, where different focal planes may render objects to look darker or brighter, interfering with the ability to consistently identify live/dead cells by their brightness/darkness.

**Figure 1:**
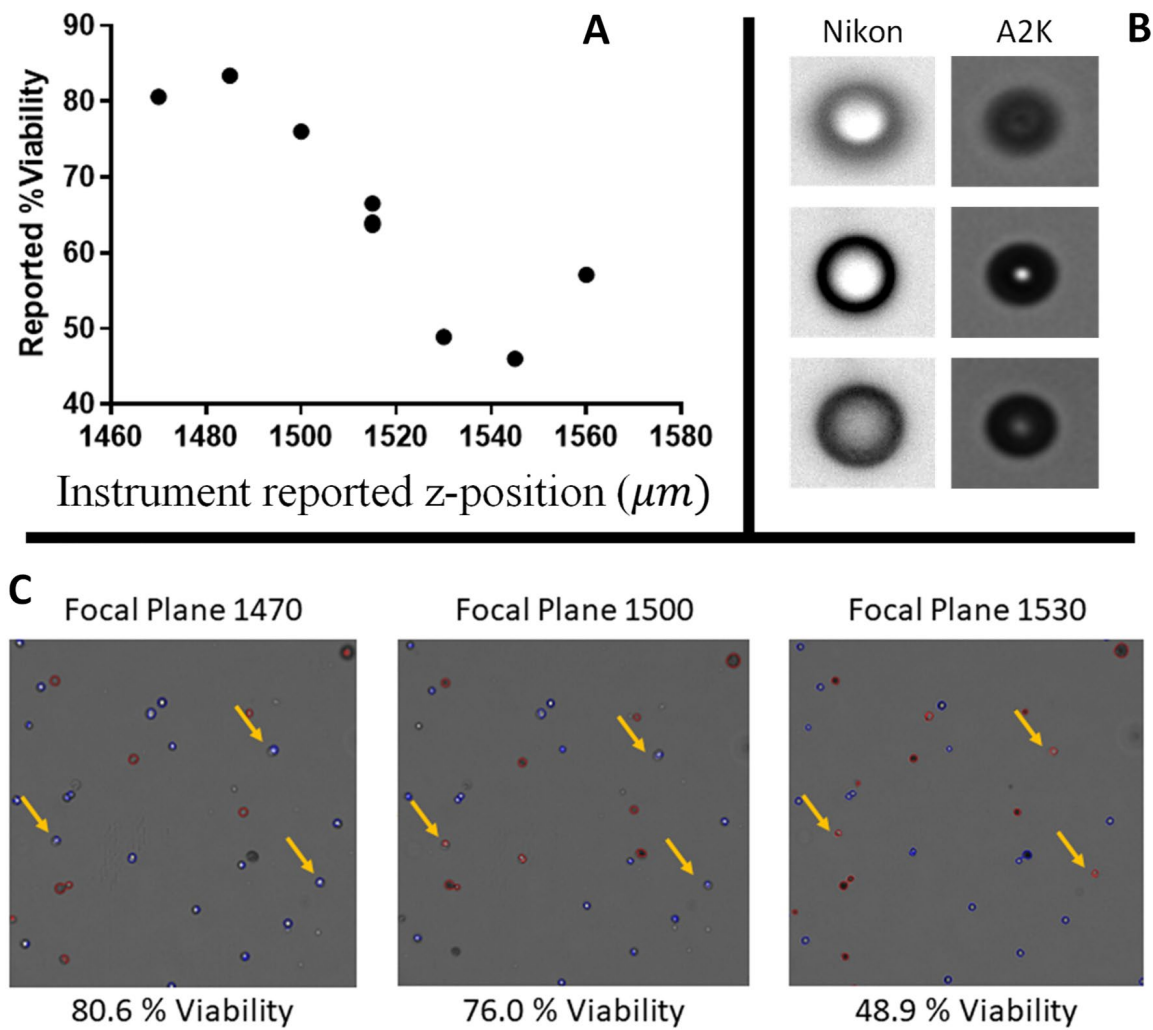
Example of variability in cell viability results for a single sample evaluated at different instrument reported z positions (focal planes), seen in panel (**A**). Panel (**B**) presents a ViaCheck 100% viability control microsphere (Bangs Laboratories Cat # VC50B) imaged on two different imaging systems (Nikon and A2K), showing the difference in the appearance of the bead as a function of distance from the reference focal plane. In Panel B: the left side are beads imaged by Nikon, top left is: − 12 μm from reference focal plane; middle left: reference focal plane; bottom left: reference plane + 12 μm; the right side are beads imaged by A2K, top right: − 75 μm from reference focal plane; middle right: reference focal plane; bottom right: reference plane + 75 μm. Panel (**C**) images represent the same field of view of the cell sample, captured at three different focal planes as indicated. Focal plane number corresponds to instrument reported z-position (μm). Red and blue outlined cells represent dead and live cells respectively as identified by the A2K software. Arrows indicate examples of single cells that are identified both as live or dead based on the focal plane of image acquisition.

Manual focus is required for each image in our measurement system, because each sample is introduced into a cell viability analyser via a disposable slide that can vary slightly in thickness and position. To ensure that measurements are repeatable, we obtain an equivalent focal plane for each sample. Instability of the measurements over fields of view and focal levels is shown in Fig. 1A with example images in Fig. 1C. Cells and cell debris remaining at the bottom of the chamber make it difficult to identify an appropriate focal plane using typical auto-focus approaches. Cells do not have features that are consistent and homogeneous, requiring different autofocus algorithms, and cell populations can undergo physical changes over time. Our approach is to use stable control materials introduced into the counting chambers with the cell samples to determine a reference focal plane.

Beads are sufficiently uniform, to the extent that bead image features have been used to reliably determine the focal plane at which the gradient around bead edges is maximized accurately and repeatably^5^. This reference material can be used in experiments where beads do not affect the measurement being made. Here we apply beads to benchmark the Z-axis of a brightfield microscope to consistently identify a reference effective focal plane (REFP) using bead image features. The concept of a reference effective focal plane (REFP) was originally defined in our previous work^5^. Benchmarking the Z-axis is an important component of ensuring reproducible images because returning to the same nominal focal plane of an instrument does not ensure that images will exhibit the same level of clarity or blur, even on a single instrument^5^. Benchmarking the Z-axis enables the formation of a focal plane coordinate system that can be used to consistently reproduce image characteristics (e.g., blur or clarity) of stable components of the systems being measured. Conceptually, this offers increased assurance that detected differences are physically meaningful and that physical differences can be detected.

Our previous work^5^ demonstrated a technique using bead image features to define a REFP, in which the bead edge gradients are maximized, and to accurately determine how far a single image lies from that reference plane. This work was performed on a single instrument and used a combination of three bead image features. Only two of the three image features generalize for use on other instruments, thus rendering the previous modeling approach ineffective on different systems. Figure 1B shows example bead images at approximately the same effective exposure and focus levels on two different instruments. The effective exposure is compared across images by measuring the average background pixel intensity. The appearance of the beads is very different across these two instruments, and in general, bead image blur metric measurements vary from instrument to instrument. To generalize our approach, we use a framework that applies an AI regression neural network, ResNet 18, on cropped bead images. This modelling approach using AI can be applied to different types of microscopes and optical systems without having to manually engineer image features for each case. We also applied ResNet 50 and ResNet 101 and obtained similar results. Other regression networks could potentially work as well. We report results for ResNet 18, since this is the least computationally intensive network we applied.

To use these reference materials, we consider deep learning approaches, previously used to determine image quality^6^ and to virtually refocus two-dimensional image data onto three-dimensional surfaces within the sample^7^. Convolutional neural networks (CNNs) have been used to estimate the focal distance over any location on the imaging slide^8,9^, to enhance image sharpness for focal plane predictions^10,11^, and for maintaining focus during bright-field microscopy^12^. Image regression using CNNs has helped to estimate reconstruction distances^13,14^, and focal correction from a single image has been done using Fourier neural networks^15^.

There are several published methods regarding assessments of image focus using deep learning. Most approaches rely on images acquired from multiple focal planes^8,9^, whereas the currently described process forms its predictions using a single focal plane. Reference^6^ considered assessments from a single focal plane, but with a different goal. They defined an 11-point scale characterizing the defocus level of an input image, where levels are spaced in increments of roughly 3 pixels of blur. They achieved a 95% accuracy, defined as the estimated blur level of an image falling within 1 level of its ground truth. This accuracy is not directly comparable to the performance of our system, which produces REFP, a continuous output of focal plane height (µm) relative to the height at which reference beads are most in focus. REFP could be mapped to a blur radius (for reference beads), but is more importantly used to characterize the focal plane in terms of its difference from a meaningful standard (i.e., the focal plane at which beads are most in focus). As a rough performance comparison, we consider using stage repeatability (± 7.5 µm) as a category width. Centering the “true category” around the ground truth REFP definition, a criterion of being within one category corresponds to being within 10 µm of the true REFP. By this the criterion, our method achieved a 98.4% accuracy.

The novelty presented in this paper lies in the methodology that improved the accuracy of predicting an image’s effective focal plane from one image acquisition. This method, which utilizes a stable bead metric for focal plane analysis, offers several advantages over recording a Z-stack and performing post-processing on a 3D image: it removes the dependence of focal plane analysis from cell image analysis, which may be unstable over time, it reduces the amount of storage and post-processing of data necessary to do analysis after the initial acquisition time, and it necessitates fewer acquisitions which reduces the amount of light exposure on the sample itself. The use of commercially available, homogeneous, and reproducibly manufactured microspheres allows the network to focus on reliable and repeatable image features rather than using image content containing cells whose features can vary with adhesion and crowding. 2. The use of cropped bead images for network training takes out the complexity of cells and cell debris, creating a more consistent training/inferencing set for the neural net models, and allows for outlier bead removal. 3. Hyperparameter optimization and averaging results after outlier removal from 10 ResNet 18 models also improves the consistency and accuracy of the prediction. This paper also presents several novel research elements related to improving the definition of the REFP, which improves the quality of the response variable in network training and improves our ability to measure network performance. In particular, we describe: (1) Optimizing the modelled location of a bead center to maximize the relationship between pixel intensity and distance from bead center; (2) Extracting bead features across the beads, and pooling profiles of these features for each combination of experiment, exposure, and field of view for each dataset. Together, these combined approaches increase the prediction accuracy of the difference between the current Z and the REFP such that 95% of predictions based on 6 beads differed from ground truth by an amount smaller than the microscope actuator Z repeatability. We further explore effects of image exposure, image normalization, and image augmentation on the performance of the network in estimating the effective focal plane.

## Data acquisition

Additional detailed information about the acquisition protocols and the optical system are described in the supplementary document. A total of 10 datasets were acquired on three instruments: Four datasets were collected on a single Cellometer Auto2000 (Nexcelom) instrument, a fifth on a different Cellometer Auto2000 instrument, and datasets 6 to 8 were collected on a Nikon Ti2 Eclipse widefield inverted instrument. These sets are outlined in Table 1. All datasets consisted of images containing microspheres only (ViaCheck 100% viability control microsphere; Bangs Laboratories Cat # VC50B), except for 2 test sets of images containing both beads and cells. The pixel size is 1.5 µm.

**Table 1:** Descriptions of ten datasets showing the ranges of the Z (focal plane) sweep and exposures, and the reference plane of highest bead gradients.

Dataset	Z range (µm)	Z collection incr. (µm)	Exposures (ms)	Ref. Z (µm)
1- A2K training	[1200, 1800]	7.5	6, 9, 12, 15, 18, 21, 24	1507.5
2- A2K test 1	[1380, 1627.5]	7.5	6, 9, 12, 18, 22, 26	1522.5
3- A2K test 2	[1380, 1620]	30	6, 9, 12, 18, 22	1500
4- A2K test 3	[1380, 1620]	7.5	6, 9, 12, 18, 22	1575
5- A2K test 4 (new lab)	[1380, 1770]	7.5	9, 12, 18	1695
6- Nikon training	[1, 25]	1	5, 10, 20, 30, 40	13
7- Nikon test 1	[1, 25]	1	5, 10, 20, 30, 40	23
8- Nikon test 2	[1, 25]	1	5, 10, 20, 30, 40	13
9- A2K test 5 (w\cells)	[1380, 1620]	7.5	12	1515
10- A2K test 6 (w\cells)	[1380, 1620]	7.5	12	1530

A2K Test 4 was taken on a different A2K instrument than the A2K training set and A2K tests 1–3.

The dataset is available from the following link: https://data.nist.gov/od/id/mds2-2993.

## Methods

**Figure 2:**
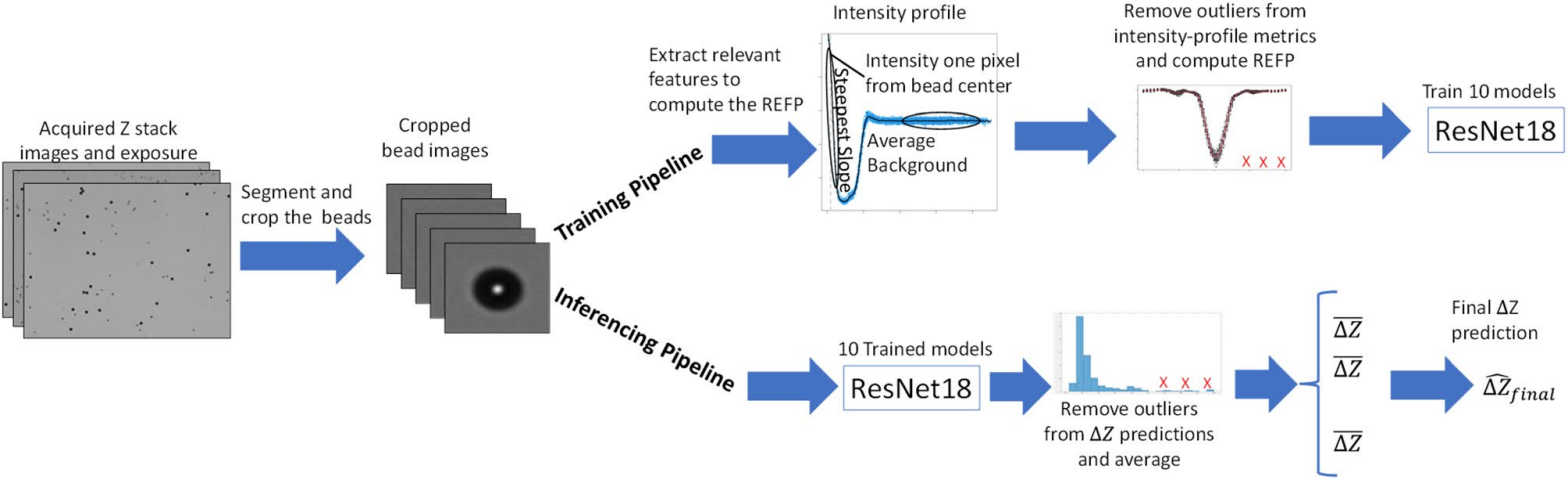
Summary plot of the method training and inferencing pipelines.

In this section, we will briefly describe each novel aspect of our work. Figure 2 provides a high-level overview of the training and inferencing pipelines of the proposed methodology.

Section “Training and inferencing pipelines” describes the neural network training and inferencing, and Sect. “REFP computation based on bead intensity profile” describes the REFP computation. Our regression network takes cropped bead images as training input and the focal plane distance Z (in µm) of each image from a REFP, which we refer to as $\Delta Z$, as training labels. New data inferenced from a trained model outputs an estimate of $\Delta Z$ for that data. It is therefore important to define the REFP for a dataset with high precision and calculate that plane using all data from a given dataset. The combination of large numbers of optimizations of our training data, which are presented here, was responsible for our AI model’s accurate $\Delta Z$ predictions.

### Training and inferencing pipelines

**Figure 3:**
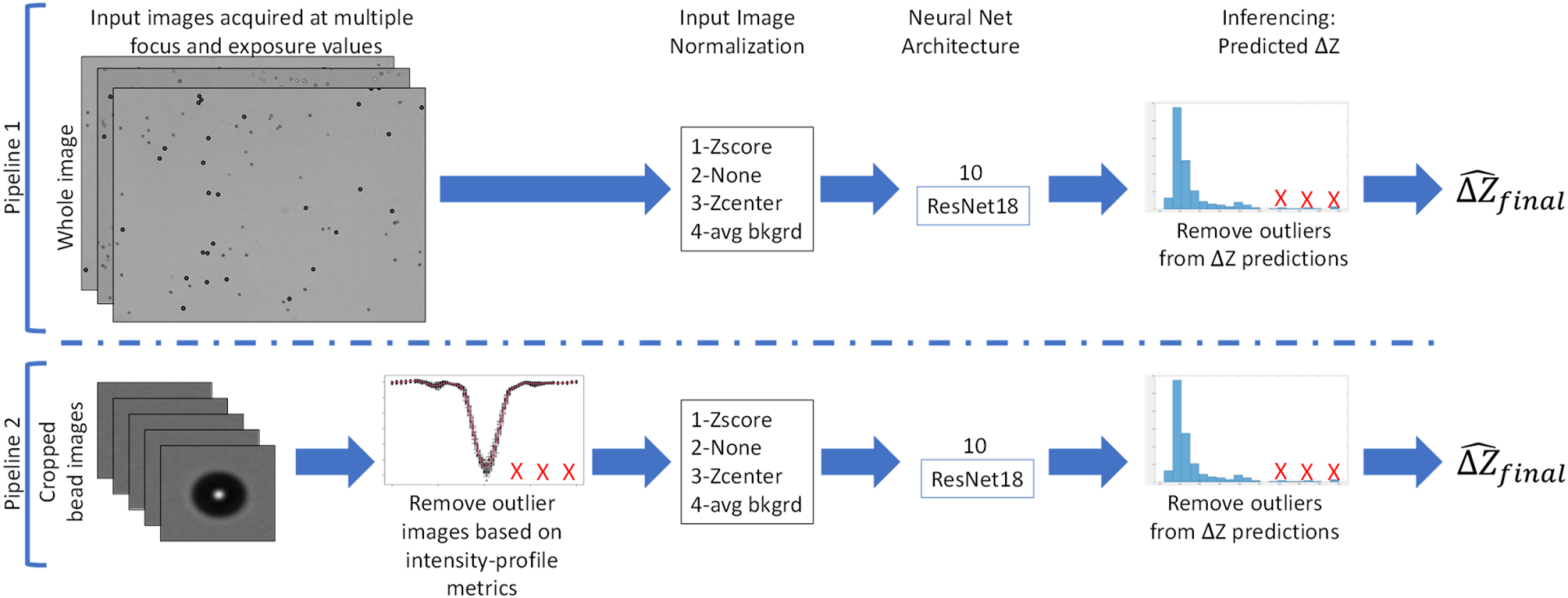
Two pipelines used to perform $\Delta Z$ prediction. Pipeline 1 uses uniformly cropped images of size 512 × 512 pixels while Pipeline 2 uses a cropped image around each bead of size 64 × 64 pixels. Pipeline 2 has an added step of outlier removal based on bead features computed on the images.

Figure 3 displays the two pipelines used to measure the differences in the $\Delta Z$ prediction accuracy across multiple factors in the training set creation. The main difference between the two pipelines is that the first pipeline uses uniformly cropped images of size 512 × 512 pixels while the other pipeline uses a cropped image around each bead of size 64 × 64 pixels. The results comparing both pipelines are showcased and highlighted in Sect. “Whole image vs cropped beads”. Pipeline 2 also has an added step of removing outlier bead images based on features computed from the bead images.

#### Bead detection

We use a simple thresholding method to segment all dark objects of a certain pixel size in the brightfield image, then search the resulting pixel clusters for appropriate bead properties. The segmentation pipeline, written in Python, is as follows: (1) Pixel clusters were found containing pixels of the lowest one percentile on the image, (2) Clusters less than 180 pixels are eliminated, (3) Clusters are checked for circularity using a circularity threshold of 0.9 for the ratio:$(4\pi \times area)/({perimeter}^{2})$, and (4) sub-images including extra background were checked for a high overall standard deviation, to eliminate sections of the background with darker shadows. To do this, the mean and standard deviation of pixel intensities in a cluster are found, and the ratio of standard deviation/mean had to be greater than 0.1.

#### Network details

We use a ResNet-18 regression model for training and inferencing. The network input are tiles of size 64 $\times$ 64 cropped grayscale bead images. Each bead is about 18 to 20 pixels (27 to 30 $\mu m$) in diameter, depending upon the image quality. Each tile is centered at the center of the bead. The network output is the focal level of the associated bead images, which we input as training labels. The $\Delta Z$ output estimates the difference in µm from the associated image to the REFP at the time of imaging. A positive value indicates the current image focal plane is above the REFP and a negative value indicates it is below the REFP.

#### Training

For each network, we set the initial learning rate at ${10}^{-4}$, the batch size at 32, and use an Adam optimizer, with a validation test every 2000 steps, using 20% of the training data for validation. We used 4 different normalization methods to apply to the input images for training and inferencing: the Z-score normalization, the zerocenter normalization, average background normalization, and no normalization. We repeatedly retrained the model using the same training set and parameters 10 times to reduce randomness or extreme values.

#### Network input

The use of cropped bead images instead of full images as input to the network increased the REFP prediction accuracy by a factor of 20. Bead segmentation is described in Ref.^5^.

#### Network input normalization

We compared 4 different normalization methods to apply to the input images for training and inferencing: Z-score, zerocenter, average background normalization, and no normalization. Using no normalization of the images led to our best results (see results section below).

$${\text{Z}}-{\text{score}}:{I}_{N}=(I-mean\left(I\right))/std(I),$$

$${\text{Zerocenter}}: {I}_{N}=\left(I-mean\left(I\right)\right),$$

$${\text{AvgBackgrd}}:{I}_{N}=I/mean\left(B\right),$$

where ${I}_{N}$ is the normalized image, $I$ is the acquired uint8 image, $mean(I)$ is the average intensity value, $std(I)$ is the standard deviation computed of intensities in the acquired image, and $mean(B)$ is the average value of background intensities.

#### Augmentation

Only random geometric augmentations were used, translation, with a [– 5, 5] pixel range and rotation, with a [− 5, 5] degree range. Reflection, jitter, and scale with a range [0.95, 1.05] augmentations were applied during training. We did not use any augmentations that changed the blurriness of the images, as the image quality of the bead tiles determines the outcome of the network.

#### Hyper-parameters optimization

We conducted a full factorial hyper-parameter optimization using the entire training set for the following parameters.

- Initial learning rate: used to scale the magnitude of parameter updates during gradient descent. Values considered between [10^−4^, 10^−2^], value selected = 1*10^−4^.
- Learning rate drop rate: number of epochs for dropping the learning rate. Values between [5, 20], value selected = 15.
- Learning rate drop value: factor (between 0 and 1) for dropping the learning rate values between [0.7, 1], value selected = 0.9.
- Mini-batch size: a subset of the training set that is used to evaluate the gradient of the loss function and update the weights. Values between [4, 32], value selected = 32.
- Validation frequency: the number of iterations between evaluations of validation metrics. Values between [500, 4000], value selected = 2000.
- Validation patience: the number of times that the loss on the validation set can be larger than or equal to the previously smallest loss before network training stops. Values between [5, 15], value selected = 10.
- L2 regularization (weight decay): Adds a regularization term for the weights to the loss function as one way to reduce overfitting. Values between [10^−4^, 10^−3^], value selected = 5*10^−4^.

**Figure 4:**
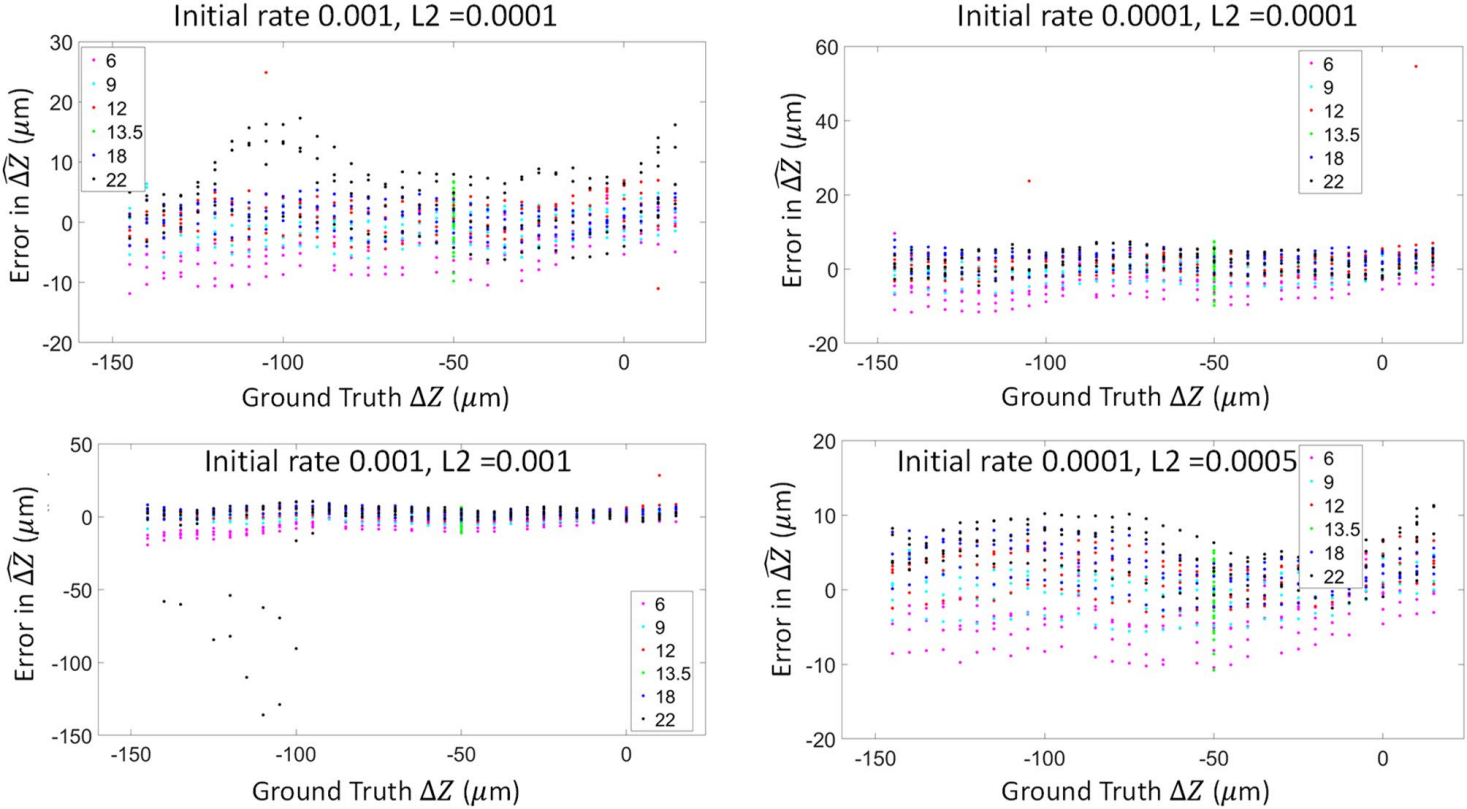
Example of hyperparameter range and optimization with L2 regularization and initial learning rate. These four plots highlight only a few examples of the hyperparameter optimization. In this figure, we show 4 outputs based on changing the two parameters (L2 regularization and initial leaning rate). The best choice is based on the overall minimized error across all images, in this case, the example on the bottom right.

The optimal value for each parameter was selected by minimizing the root mean squared error (RMSE) of the validation test (Fig. 4). This optimization helped select the optimal parameters that will yield the best predicted accuracy on the test dataset, which will maximize the generalizability of the trained model.

#### Inferencing with multiple trained models

The network output is a prediction value of the distance to the reference focal plane for the image. We inference bead images using each of the 10 trained ResNet 18 models and remove outliers for each based on the predicted $\Delta$ Z for each bead. Then we averaged the remaining $\Delta$ Z values to obtain a mean value per model, $\overline{\Delta Z },$ for each of the 10 trained ResNet 18 models, after which we average the 10 $\overline{\Delta Z }$ values to compute ${\widehat{\Delta Z}}_{final}$.

#### Importance of outlier removal

The hypothesis for this work is that most beads will be sitting correctly on the plate and have similar focal plane. The outlier removal process, based on interquartile range (IQR) and further described in the Supplementary Document, is intended to filter out incorrectly positioned beads so that subsequent inferencing will be done on beads sitting at similar focal planes, which helps improve the accuracy of the predicted ${\widehat{\Delta Z}}_{final}$ value.

### REFP computation based on bead intensity profile

**Figure 5:**
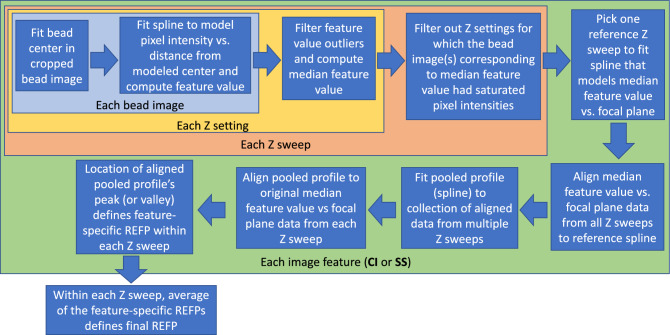
Flowchart overview for the process of defining the REFP for each Z sweep.

This section describes the process for defining REFPs for a given experiment on a given microscope, whose accuracy plays a large part in achieving good results for our AI model. A high-level overview of this process is given in Fig. 5. The Supplementary Document provides additional details beyond those summarized in this section. The process of defining the REFP relies on the relationship between effective focal plane and image features evaluated from cropped bead images. The relationship between nominal focal plane and effective focal plane can potentially differ from one microscope to another, or even on the same microscope over time. Here we describe the process of extracting two specific bead image features that can consistently be used across different instruments to define an REFP for a given optical system. This process can be applied to brightfield microscopy images of beads. We assess the stability of the relationship between image features and effective focal plane for a given microscope across time, across two microscopes of the same make and model, and across microscopes made by two different manufacturers.

### Bead center location optimization

**Figure 6:**
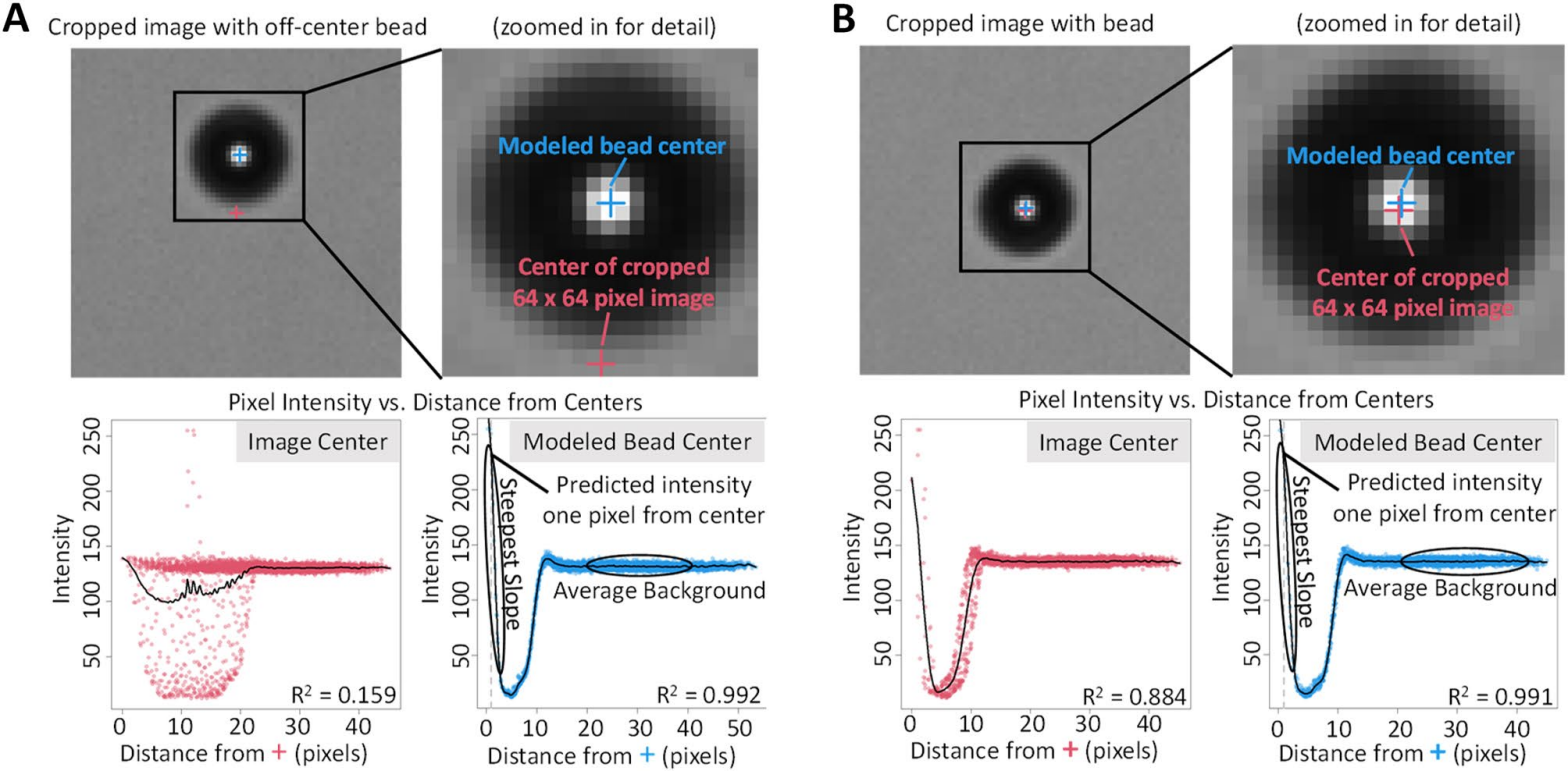
(**A**) Example in which the center of a bead is not near the center of the cropped image. (**B**) example in which the center of a bead is near the center of the cropped image; For each example the image in the top left displays the 64 × 64 pixel cropped bead image and a zoomed-in (32 × 32 pixel) view to its right, where we show the difference between the modelled bead center (blue) and the image center of the original 64 × 64 image (red). For each example we plot the relationship between pixel intensity and distance from the 64 × 64 image center (red) and the relationship between pixel intensity and distance from the modelled bead center (blue). Features are extracted from the cubic spline fit to the blue data as part of the process for determining the REFP. The considered portions of the spline fit (determined by specifying a given range of distances from center) are circled for two such features, steepest slope and average background.

The REFP is defined by bead image features that are dependent upon having a good approximation of the location of the center of a bead, so we describe how we accurately achieve that location. Following bead segmentation, we crop a 64 × 64 pixel region centered around the bead’s segmented mask such that the bead center, as computed from the segmented mask, is located at (32,32). The underlying physical reference bead is far more continuous than the pixels used to describe it, and the center of the bead is likely to fall in the middle of a pixel. We therefore conduct an optimization over continuous X,Y coordinate space to identify the location of bead center. This optimization also accommodates instances in which the bead is substantially off center in the 64 × 64 image, such as when the bead is near the edge of the field of view. The optimization is conducted to minimize the sum of squared residuals from a cubic smoothing spline fit to model the relationship between distance from bead center and pixel intensity. The illustration in Fig. 6 shows the effect of optimizing the bead center location on the fitted profile (bottom right panel with blue data points for each of the two examples in Fig. 6) as compared to using the center of 64 × 64 cropped image (bottom left panels with red data points). In particular, the optimized profile, where the center of the bead does not have to coincide with an integer pixel location, shows a higher intensity at distances near 0, representing the brightness of the bead center, compared to the profile that treats the center of the cropped bead image as though it is the center of the bead. The left panel of Fig. 6 illustrates the importance of modelling the bead center when the bead is far from the center of the cropped image, which can occur, for instance, if the bead is near the edge of the field of view. The example on the right illustrates the importance of even minor (i.e., sub-pixel) adjustments to the bead center location. Even though the two considered centers are only roughly half a pixel apart, the effect on the modelled center intensity, as seen in the difference between the spline fit at x = 0 for the red (about 210) and blue (about 260) profiles for the bead on the right, is roughly 20%.

#### Metrics for estimating the REFP

Once the bead center and the intensity vs. distance from center profiles have been evaluated for all beads in a FOV image, we extract two features from the intensity profile for each bead, steepest slope, and center intensity. The steepest slope feature is given by the derivative value farthest from 0 evaluated from the smoothed spline fit to pixel intensity vs. distance from bead center. The center intensity feature is provided by the value of the smoothed spline at 1 pixel from the center. Each of these features are normalized by the average background pixel intensity value, which is computed as the mean of the fitted spline values over the range from 20 to 40 pixels from the center. Background normalization is performed to negate the effects of different exposure durations or brightness levels and spatial variability in the brightness across a given field of view. These aspects of the smoothed spline are illustrated in bottom right plots of Fig. 6. We refer to the background-normalized steepest slope and center intensity features as **SS** and **CI**, respectively.

#### Pooled profile

For each combination of experiment, exposure, field of view, and Z, we compute our two metrics, **SS** and **CI**, for each bead. We then perform the automated outlier removal (described in Section 2.1.3 in Supplementary Document) for each feature and compute the median feature value among the remaining beads. We also record how many pixels are fully saturated in each bead image. Then we fit a cubic spline to model the relationship between median feature value and Z for each combination of experiment, exposure and FOV. We use these spline fits to align all data (allowing for an additive shift in focal plane and a multiplicative rescaling in feature value) with the data from one chosen combination of experiment, exposure, and FOV. This creates a common effective focal plane (i.e., Z-scale). We then fit a cubic smoothing spline to the collective, aligned data to construct a pooled profile describing the relationship between feature value and effective focal plane.

#### Feature-specific REFP

**Figure 7:**
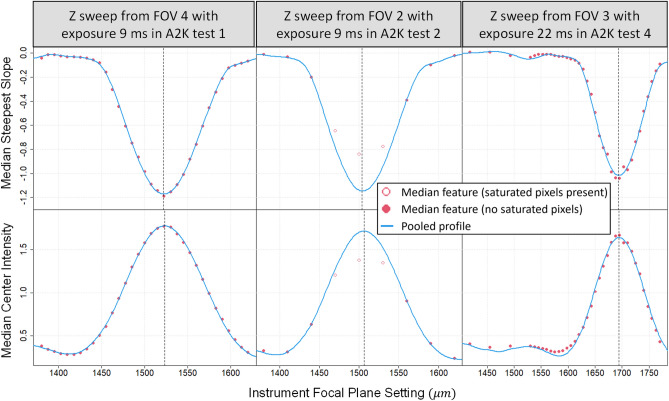
Examples of aligning the pooled profile to the median feature values for three separate Z sweeps. Top panels use the steepest slope feature and bottom panels use the center intensity feature. Red points depict median feature values. Hollow points indicate that at least one pixel was fully saturated in the median bead image(s) (sorted by feature value). Solid points indicate that no pixels were fully saturated in the median bead image(s) (sorted by feature value). Blue curves depict the pooled profile aligned to the solid points (i.e., the unsaturated medians). Vertical dashed lines depict the feature-specific REFP for each example Z sweep.

The pooled profile is then fit (allowing for an additive shift in focal plane and a multiplicative rescaling in feature value) to the median feature values and original Z values for each combination of experiment, exposure, and FOV. Feature-specific REFPs are given by the Z value at which **SS** is minimized and the Z value at which **CI** is maximized. Additional details for this process are provided in the Supplementary Materials. Plots showing the fit of the pooled profile to the median feature values for Z sweeps from a few combinations of experiment, exposure, and FOV are shown in Fig. 7. The center panels of Fig. 7 depict the benefit of using a pooled profile, where fewer data points are available for this particular set. This smaller set of images were collected at wider increments of focal plane, and images collected near the REFP had saturated pixels. Results from focal planes for which most beads had saturated pixels (indicated by hollow points in Fig. 7) were excluded from the analysis because pixel saturation can substantially impact the steepest slope and center intensity metrics. By using the pooled profile, we are still able to get a precise characterization of the REFP from the six focal planes for which saturation did not affect the median feature value. Additionally, the three datasets shown illustrate the stability of the shape of the relationship between focal plane and feature value, while the variability in peak locations across the three datasets shown illustrates that the nominal focal plane on the instrument is not an adequate indicator of the effective focal plane, even across experiments on a single microscope.

#### REFP computation

After completing this process using features **SS** and **CI**, respectively, a final REFP is defined for each combination of exposure, FOV region, and experiment as (REFP_**SS**_ + REFP_**CI**_)/2. These REFP values are the response variable used for training and testing the AI network.

## Results

### Whole image vs cropped beads

**Figure 8:**
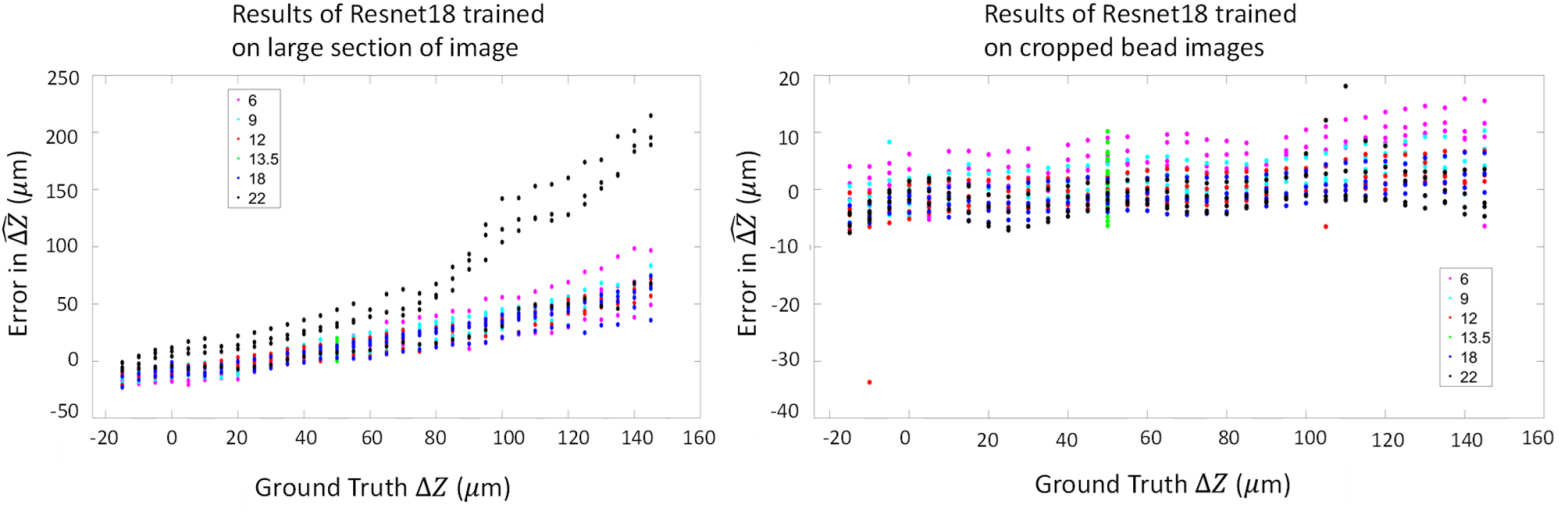
Results of inferencing on A2K test 3 with whole image tiles (left) and cropped bead tiles (right), on a network trained with images from the Nikon training dataset. The x axis shows the depth levels of the Z sweep in dataset 3, and the y axis shows averaged errors for predicted ${\widehat{\Delta Z}}_{final}$ values from 10 trained ResNet 18 models sorted by colours with respect to the exposure level.

Cropped bead images have more consistent image features than our full images containing cells. Figure 8 shows a large increase in accuracy when training with only the cropped bead images compared to training with larger image tiles (512 × 512) that included both beads and cells. These results also include the improvement of the training set by removing outlier beads before training. The inferencing results shown use A2K test 3, either with whole image 512 × 512 tiles (left) or cropped bead 64 × 64 tiles (right). These results correspond to the average of 10 ResNet 18 networks trained on images from the Nikon training dataset in Table 1. It is obvious that cropping the beads to create the training sets has contributed to a major improvement in prediction accuracy. In this experiment, most errors after training on the cropped beads are less than 10 µm. When training on large sections of the images, the ΔZ predictions became substantially biased as the magnitude of the ground truth ΔZ increased.

### Image normalization

Images were collected across a range of exposure settings. Within the linear dynamic range of the charge-coupled device (camera), exposure is expected to have, on average, a multiplicative scaling effect on pixel intensity. We expect the relationships between image features and effective focal plane to be robust across this range of exposure. Traditional image processing often uses Z-score normalization where pixel intensities are centered to have a mean of 0 and scaled to have a standard deviation of 1. Here we investigated the effects of normalization by centering and scaling separately using a 2 × 2 factorial design. That is, we considered (1) neither centering nor scaling; (2) centering, but not scaling; (3) scaling by the average background intensity, but not centering; and (4) centering and scaling, in the traditional Z-score fashion.

**Figure 9:**
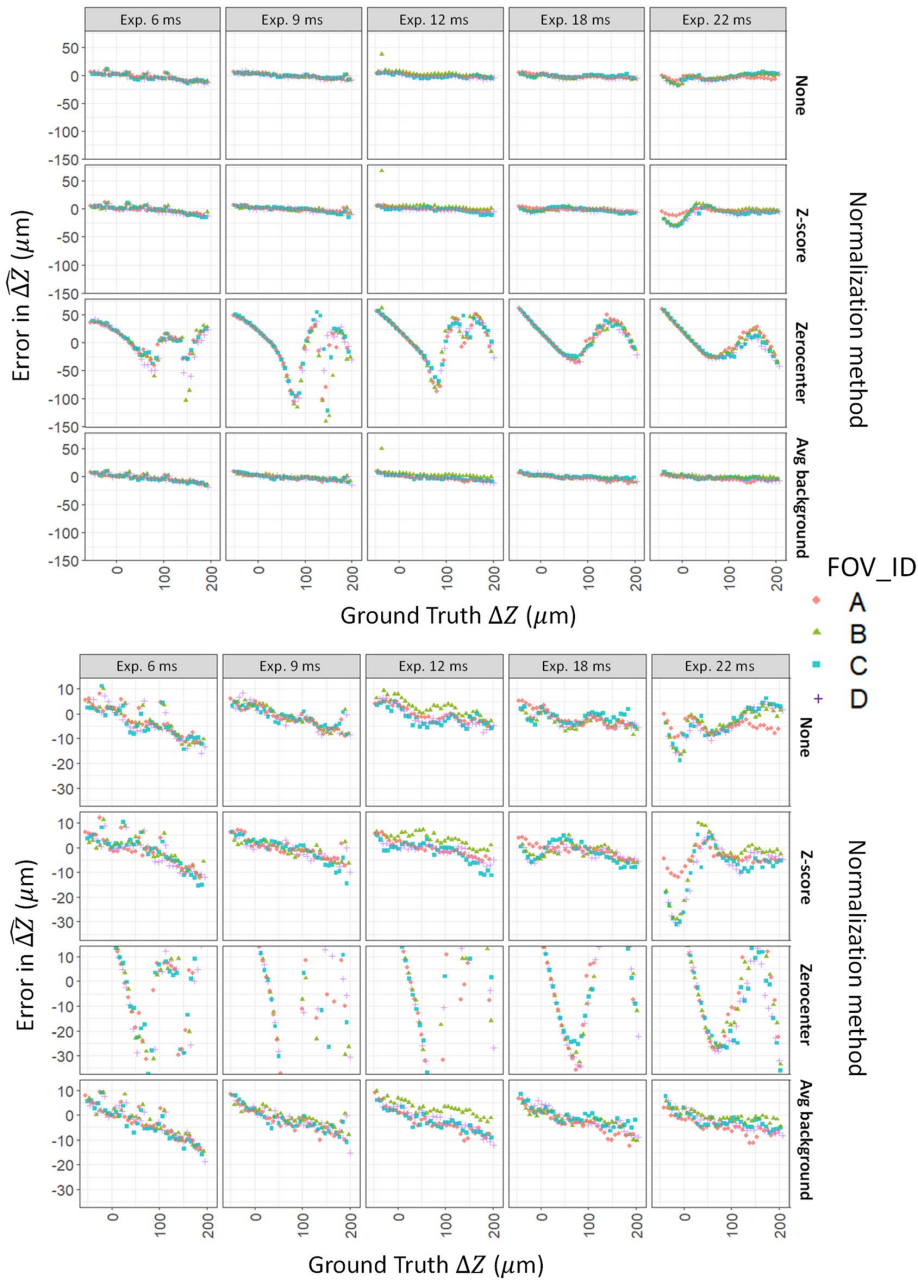
Results when using different normalizations: for both plots: (top row) without normalization; (second row) Z-score normalization; (third row) Zerocenter; (bottom row) dividing by average background. The y axis in the top plot includes all ${\widehat{\Delta Z}}_{final}$ residual values. The y axis in the bottom panel is narrowed to better facilitate comparison between results from normalization approaches other than Zerocenter. The x axis shows the depth levels of the Z sweep in A2K test 3, and the y axis shows averaged errors for predicted Z values from one of the trained ResNet 18 models. Results without normalization worked as well as any of the other normalization methods.

Network training was performed using cropped bead images from the Nikon training dataset, performing bead outlier removal based on the steepest slope (SS) metric values, and then testing on cropped beads from A2K test 3, also following outlier removal. The results are shown in Fig. 9 for all four normalization methods. The results show that doing no normalization performed well, and similarly to the method that includes scaling but not centering, and that centering without scaling performed substantially worse. The trend in residuals seen in Fig. 9 for centering without scaling shows that the network had substantial systematic biases in its REFP prediction. Interestingly, both the Z-score normalization and no-normalization methods show signs of slight bias among images taken just below the REFP at exposure 22, while the scaling only normalization does not. The bottom panel of Fig. 9 shows a slight drift (~ 20 µm) in REFP residuals across the focal plane sweep covering about 250 µm for the no normalization, Z-score, and scaling without centering methods. This drift is roughly at the scale of the actuator repeatability (~ 7.5 µm), and we did not further investigate the cause of show this drift for this dataset bias.

### Number of models and number of beads

**Figure 10:**
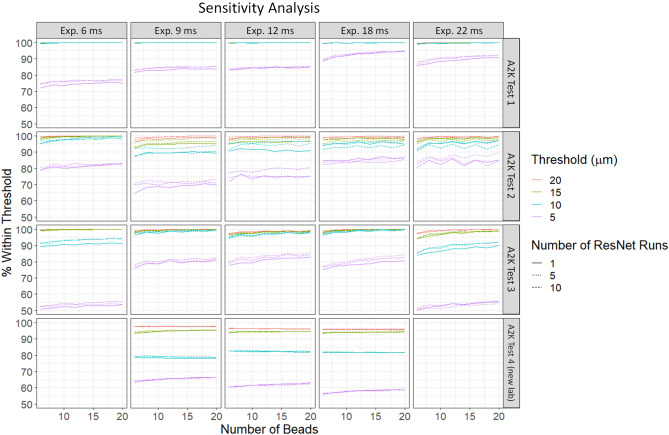
Sensitivity analysis of number of models and number of beads necessary to achieve a demanded level of prediction accuracy: the x axis shows number of beads along the bottom and exposure levels (ms) along the top. For each dataset and exposure combination, we show the percentage of beads within four different sets of threshold levels. The plots show little change in the percentage within threshold as the number of beads is increased above six. A2K test 4 was taken on a different A2K instrument at a different lab than the other A2K sets and does not contain images at exposures 6 ms or 22 ms.

We combined the output of 10 different Resnet 18^16^ models trained on the same dataset to compute the final Z predictions. The idea is to reduce variability in the AI predicted values that may arise from any one instance of a trained network. Because each Resnet 18 model begins with randomly assigned coefficients, the final weights of the 10 Resnet 18 models are slightly different after training, even when the same images are used to train each model. To examine the effect of using multiple models, we examined how model performance changed when using 1, 5, or 10 Resnet 18 models. For each bead image, we used the median of the AI predictions across the Resnet 18 models. We inferenced from 6 to 20 beads with each Resnet model, averaging output results for each bead, and then removing outliers using interquartile filtering. This resulting average was compared to the actual effective focal plane (i.e., the difference between the nominal focal plane value on the microscope and the REFP, as determined following the approach described in Sect. “Methods”). Figure 10 shows how often the obtained results were within various thresholds. The top three rows correspond to test data acquired on the same microscope as the training data, while the bottom row shows the performance when applied to images gathered on a different microscope of the same make and model. (No images were collected at exposures of 6 ms or 22 ms on this second microscope, which is why the corresponding panels for A2K test 4 in Fig. 10 are blank). In all considered scenarios, predictions based off a single Resnet 18 model applied to six bead images were within 5 µm of the ground truth at least 50% of the time and above 70% in 13 out of 15 combinations of experiment and exposure where training and test images were acquired on the same microscope. Across all scenarios, at least 80% of predictions were within 10 µm using only six beads. For test images from the same microscope, 14 out of 15 combinations of experiment and exposure produced predictions that were within 10 µm at least 90% of time in all considered scenarios. It is recommended to retrain on the data for each instrument to achieve optimal performance of the model.

The results generally show improved performance as the number of Resnet models or number of beads increase, except for the testing data from a different microscope. This likely indicates that the observed differences between AI output and the REFP scale for A2K test 4 is due to a slight bias between the microscopes, rather than variability among bead images or Resnet models.

The number of Resnet models applied in practice should be chosen with the cost–benefit trade-off in mind (additional computing time or resources versus potential for moderate improvement in algorithm performance). The ideal number of beads will ultimately depend on the sensitivity of the end-use image characteristics to focal plane and the sensitivity of the cell population to beads. However, these results show strong performance even when using only six beads and a single Resnet 18 model.

### Test on different instruments

**Figure 11:**
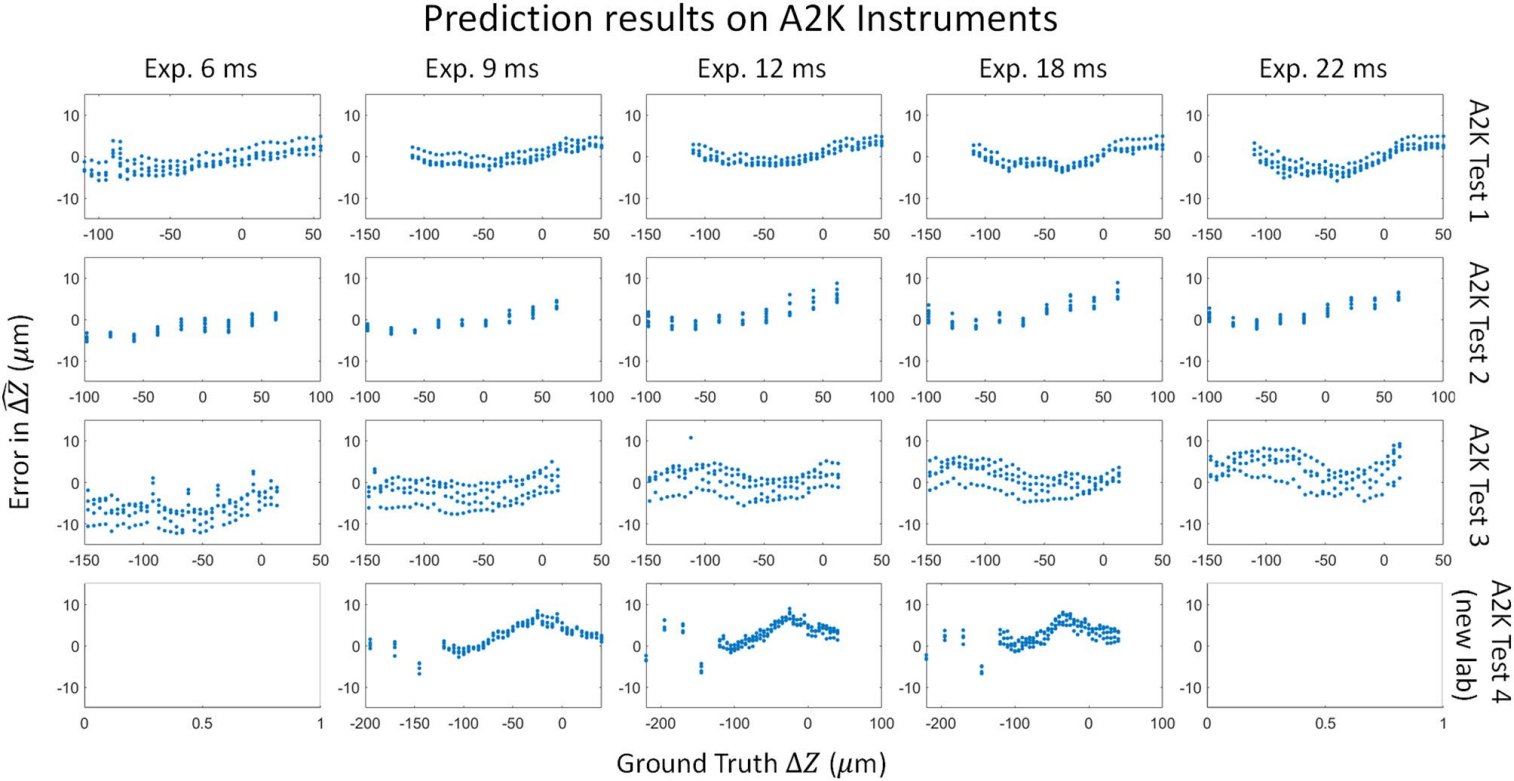
Plots of averaged errors vs. ${\widehat{\Delta Z}}_{final}$ sweep for A2K tests 1–4, using a model trained on the A2K training data. Each point in the plot is the average prediction from 10 models after outlier removal. Images for A2K test 4 were acquired at a different lab; these data did not include exposures 6 or 22. Exposure time units are ms.

**Figure 12:**
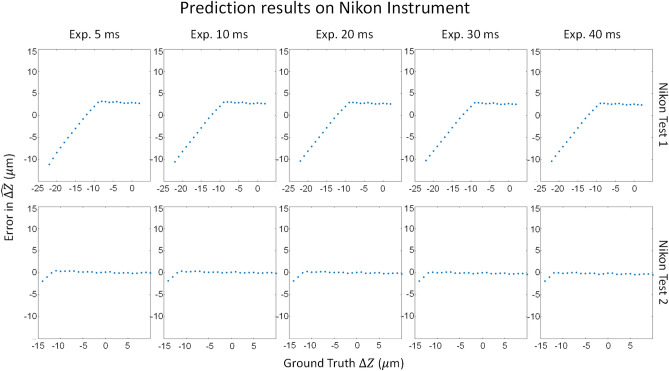
Plot of averaged errors vs. ${\widehat{\Delta Z}}_{final}$ sweep for Nikon tests 1 and 2, using a model fit to the Nikon training dataset. Each point in the plot is the average prediction from 10 models after outlier removal. The training dataset included effective focal planes as low as − 12. Nikon test 1 was acquired with a higher reference focal plane, so the lowest z value data was not represented in the training data of the model.

Figure 11 shows results from all A2K test datasets and Fig. 12 shows results from the Nikon test dataset as described in Table 1. Because each dataset has its REFP at a different nominal focal plane, to compare Z sweeps from different sets we use effective focal plane, computed as distance from REFP ($\Delta Z$), instead of the instruments’ nominal Z settings.

The pipeline to compute the final $\Delta$ Z prediction is the same for all datasets and instruments:

1. Segment beads for each acquired image (Z, and exposure pairing) and crop to 64 × 64 image tiles for the A2K and 128 × 128 for the Nikon.
2. Remove outlier beads using interquartile range (IQR) filtering based on the Steepest Slope metric. (See Supplementary Document for further details.)
3. Inference bead images using each of the 10 trained ResNet 18 models and remove outliers for each based on the predicted $\Delta$ Z for each bead.
4. Average the remaining $\Delta$ Z values to obtain $\overline{\Delta Z }$ for each of the 10 trained ResNet 18 models
5. Average the $\overline{\Delta Z }$ values from the 10 trained ResNet 18 models to compute ${\widehat{\Delta Z}}_{final}$.
6. Evaluate the errors from our models by comparing ${\widehat{\Delta Z}}_{final}$ with the evaluated difference between Z and corresponding REFP values for each set, as listed in Table 1 and as computed in the Supplementary Document.

Inference for A2K tests 1–4 was performed with a model trained from the A2K training data, which was acquired across a $\Delta Z$ range of − 307.5 $\mu m$ to 292.5 $\mu m$. Inference for Nikon tests 1 and 2 was performed with a model trained from the Nikon training data, which was acquired across a $\Delta Z$ range of − 12 $\mu m$ to 12 $\mu m$. For all data within the $\Delta$ Z range of our large training sets, errors on the final ${\widehat{\Delta Z}}_{final}$ prediction were less than the target value of 7.5 $\mu m$, which corresponds to the mechanical uncertainty of the Z actuator. This is true even for A2K test 4 (from a second lab), in which more individual bead predictions were lower.

The results of testing on the Nikon images showed consistent very low errors, equivalent to the accuracy from the A2K model, even though the bead gradients are not as sharp as in the A2K images, which can be seen in the sample bead images in Fig. 1. Within the range of Z values of the Nikon training set, all computed errors in predicting $\Delta Z$ on Nikon bead images remained less than 10 $\mu m$, as shown in Fig. 12. Nikon test 1 images were acquired across a different range of effective focal planes than the Nikon training set as shown in Table 1. Although both datasets were collected across a Z range of 1 $\mu m$ to 25 $\mu m$, Nikon test 1 has a REFP around Z = 23 $\mu m$ while the Nikon training set has a REFP at 13 $\mu m$. Thus, Nikon test has an effective focal plane ($\Delta Z)$ range of -22 $\mu m$ to 2 $\mu m$, while the Nikon training set has an effective focal plane range of -12 $\mu m$ to 12 $\mu m$. We can see from Nikon test 2, which also has its REFP at 13 $\mu m$, that the network works within a 5 $\mu m$ error range when operating within the range of effective focal planes included in the training set. Nikon test 2 has a similar $\Delta$ Z sweep range as the training set.

### Testing the pipeline on images with cells and beads

**Figure 13:**
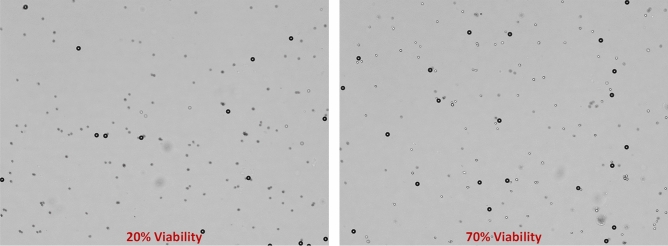
Example images with beads and cells, left is 20% viability and right is 70% viability.

We tested this methodology on images with cells and beads and there were no implications on REFP prediction accuracy with the presence of cells. We acquired two datasets with Z sweep values ranging between [1380, 1620] $\mu m$ with a mix of beads and cells, one dataset has low viability (less than 20%) and another dataset with high viability (70%) as shown in Fig. 13. These datasets are referred to in Table 1 as A2K Test 5 (w/cells) and A2K Test 6 (w/ cells). Viability was determined using the NucleoCounter NC-3000 (ChemoMetec, Lillerod, Denmark) with acridine orange and DAPI (4', 6-Diamidine-2'-phenylindole dihydrochloride), a well-established and commonly used viability analysis technique in the cell therapy field. Both datasets were acquired at exposure 12 ms.

**Figure 14:**
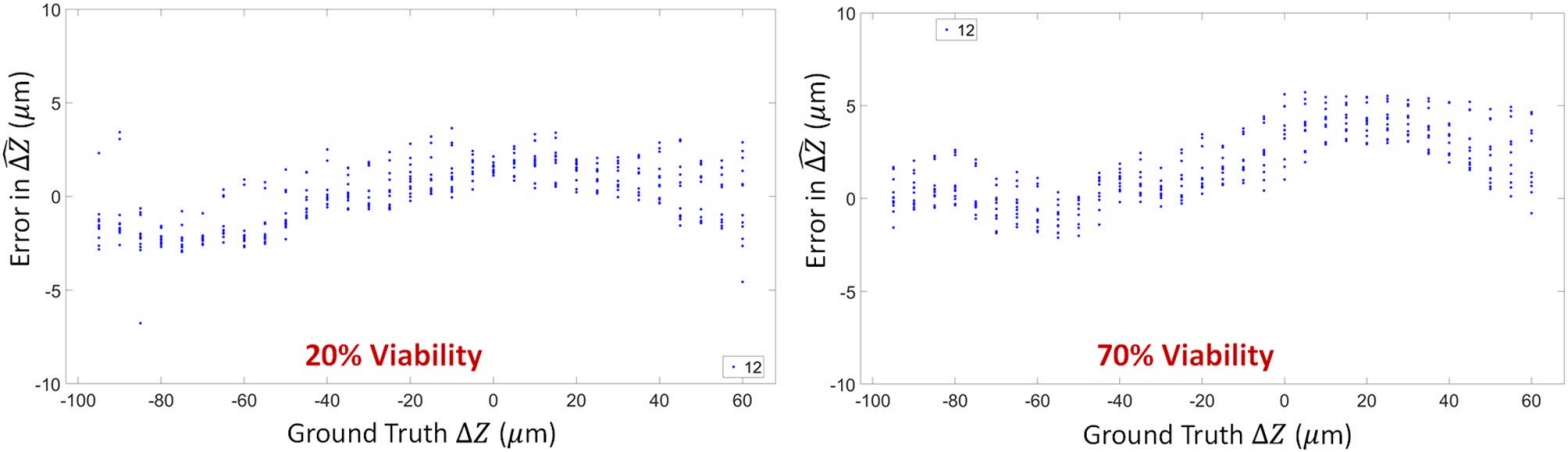
Prediction Results of the entire pipeline when applied to datasets with cells and beads. For all images in both test sets, the errors in predicting the appropriate REFP was less than 10 µm.

The prediction results of the pipeline are shown in Fig. 14. The pipeline worked well even with confluent cells present in the dataset. Only 6 beads are necessary to run this calculation as well, and the prediction was below the required tolerance of 7.5 µm.

## Discussion

Many of the most commonly used cell count and viability analyzers utilize image-based methods to obtain live and dead cell counts, and the counting algorithms associated with these instruments rely on the acquisition of high-quality images to ensure that count is robust and accurate. As a result, cell-based imaging measurements often vary with image quality. By defining an image-based focal plane, researchers or analysts can specify an effective focal plane on which to take high quality measurements in a manner that enables those measurements to be reproduced at different times and on different instruments. Such a measurement is useful so that cell-based assay measurements that depend upon image quality can be compared across different instruments and imaging conditions.

We used beads as a reference material to help improve focal plane stability within a given instrument and comparability across different instruments. We expanded our prior work to make our modelling approach easy to implement on a large variety of instruments and demonstrated its effectiveness and accuracy on instruments on which the bead images clearly look different. We improved on previous AI work in this field by applying several new concepts while creating our training data. To enable the network to focus on image features of our reference beads, image regions containing isolated beads were cropped out and outliers removed. These two updates presented a more consistent set of (bead) image features to the network than what typically results from heterogenous cell populations. We have already created a segmentation technique and morphological filtering to segment the beads out of the cell population^5^, so the network will only see the bead images as input for the predictive model.

We proved that the methodology presented in this paper can achieve high accuracy in predicting the position of a given image’s focal plane relative to a REFP with high repeatability, as good as the stage uncertainty level. In order to achieve this level of accuracy, we optimized our networks with respect to image normalization and several other network hyperparameters. In addition, it was essential to define a REFP for each dataset using all the data in that set, in as precise a way as possible. We have also shown that an instance of the AI model fit to a single data collection from one instrument produced accurate focal plane characterizations for other data collections on that same instrument and for a data collection on another instrument of the same model from the same manufacturer. This new methodology can also be re-trained to a different microscope with different optics without needing to manually engineer image features.

The main limitations of this methodology are the two features used to define the REFP: The steepest slope and center intensity image features. These features were selected after observing that pixel intensities in the bead centers were sensitive to focal plane. In particular, we noticed individual bead centers appeared to achieve their brightest centers nearly in unison within a narrow range of focal planes. We expect our approach for defining an REFP to work well for other optical systems in which beads exhibit this general behaviour. If there is substantial variability in the depth of beads within a sample, the REFP may not be clearly defined. Additionally, for systems where bead centers do not tend to become brightest near a useful focal plane, the image features we have chosen may not be suitable for defining the REFP. However, as long as bead images are roughly radially symmetric about their center, a cubic spline should suitably reflect relevant intensity information from bead images, and one could simply select other features of the spline to define a REFP. An additional limitation is that the desired focal plane for a given experiment must not be so far from the REFP that the beads do not appear in the image.

## Conclusions

We have demonstrated our method for establishing an image-based focal plane coordinate system using bead features, so that cell-based assays that depend upon image quality can be better compared across different datasets and instruments. We increased the accuracy of focal plane prediction over past performance by cropping bead images, removing bead outliers, and using more information to refine the reference effective focal plane used during training and evaluation. Our previous work to define the effective focal plane intensity coordinate system was dependent upon the consistency of particular bead image features in order to maintain high prediction accuracy. Using a deep learning network to capture image features frees us from manually engineering image features that may only work for a particular instrument. Instead, the same general AI training process can be performed on each instrument for which the model is needed, and we have shown this results in high accuracy for two different instruments with substantially varying bead images. As future work, we would like to explore the usage of different reference materials (other than beads) to further improve accuracy and minimize cell to bead interactions.

### Supplementary Information

Supplementary Information.

## Data Availability

All datasets used in this research is available online at: https://data.nist.gov/od/id/mds2-2993.
