# Supplementary material for: Establishing a reference focal plane using convolutional neural networks and beads for brightfield imaging
Source: Sci Rep. 2024 Apr 2;14:7768. doi: 10.1038/s41598-024-57123-w (PMC10987482; doi:10.1038/s41598-024-57123-w)
Supplement: Supplementary file 1 — Supplementary Information. [file 41598_2024_57123_MOESM1_ESM.docx]

Establishing a Reference Focal Plane Using Machine Learning and Beads for Brightfield Imaging

Supplementary Document

# DATA ACQUISITION

## Images with only beads

The bright field images are acquired both with and without cells present in the images. All of our image sets contain beads. The Bangs 100% ViaCheck viability control beads (Bangs Labs, VC50B, stored at 4°C) were used to benchmark image focus. The 100 % ViaCheck beads were selected due to their ability to be easily distinguished from Jurkat cells (approximately twice the diameter) and their bright center spot, which can be used to aid in manual focusing. For obtaining datasets with beads only, 50 μL of bead solution was mixed with 50 μL of 0.4% trypan blue (Gibco #15250-061) to obtain a final bead suspension in 0.2% trypan blue concentration. For bead only studies, following sample injection, the inlet and outlet ports of the slides were sealed with an epoxy resin (Devcon Flow-Mix 5-Minute Epoxy, Product ID 20445-6PK). The epoxy was mixed for two minutes using a pipet tip and then a drop of epoxy, sufficient only to seal the inlet and outlet ports. Properly sealing the slides ensured that there was minimal bead movement and evaporation over the time course of each study (approximately 2 - 3 hours). The sealant was allowed to set for 15 minutes prior to the start of imaging. The sample was allowed to settle for at least 30 seconds prior to the start of each imaging set.

## Images with beads and Jurkat cells

For obtaining datasets containing both beads and cells, Jurkat cells (Jurkat, Clone E6-1, ATCC TIB-152) were cultured in HyClone RPMI 1640 media (HyClone # SH30096.01 ) supplemented with 10% Fetal Bovine Serum (ATCC # 30-2020) and Glutamax (Thermo Fisher # 35050061 ) Cells were maintained in suspension culture in T-75 flasks (Corning # 1012611) at 37°C and 5% CO2 and used between passage 16 and 30. Cells were prepared at concentrations of approximately 2 x 106 cells/mL for stock cell solutions.

## Loading samples on the first instrument

The first instrument is the Cellometer Auto 2000 (A2K) with the SD100 slides from Nexcelom Bioscience. The SD100 slide is inserted into the imaging chamber of the A2K. The A2K automatically captures and saves four images (Fields of View or FOV) per sample at each desired focus and brightness setting (although only a single image is needed for our method described here). Images were captured over a full range of focal planes (1380 to 1620 μm height relative to the instrument optics) in increments of 7.5 μm. Table 1 outlines the datasets we collected, both for creating a reference dataset and as test sets, describing the exposure and Z sweep ranges. Data containing a full focal range sweep were acquired in five separate studies conducted on different days for replication, including a set containing cells and beads, and a set taken on a separate A2K instrument. Resulting images contain 1280 x 960 pixels, with beads typically having a diameter of 18 - 19 pixels dependent upon the clarity of the bead (1 pixel correspond to 1.5 μm in x,y).

## Loading samples on the second instrument

The bead-based approach for identifying a reference focal plane was applied to a different microscopy system, the Nikon Eclipse Ti2 Microscope. The microscope was configured with a Hamamatsu digital camera (C11440, ORCA Flash 4.0LT) and images were acquired with a 10X objective (CFI60 Plan Fluor Phase Contrast DL 10x Objective Lens, N.A. 0.3, W.D. 16mm, F.O.V. 25mm, Ph1) in brightfield mode, over a range of exposure settings (10 ms to 30 ms). For each exposure setting, a z-stack was captured of a single field of view with a step size of 1.69 µm (z-stack captured 136 images). Bead samples were prepared by injecting 10 μL of bead solution into an NC-Slide A-8 8-chamber sampling slide (Chemometec Cat #942-0003) Approximate final bead concentration for Nikon imaging studies was 1 million/mL.

# MEthods

## Reference effective focal plane computation based on bead intensity profile

This subsection describes process for evaluating reference effective focal planes (REFP) for a given z-sweep on a given microscope. It relies on the relationship between focal plane and image features evaluated from the cropped bead images in the previous section. The relationship between image feature and focal plane can potentially differ from one microscope to another, or even on the same microscope over time. Here we describe the process of extracting image features and estimating their relationship with focal plane on a given camera. The process described here can be applied any given live-cell-imaging microscope. We later assess the stability of the relationship between image feature and focal plane for a given microscope across time, across two microscopes of the same make and model, and across microscopes made by two different manufacturers.

For each cropped bead image from a given microscope, the location of the center of the bead is modelled based on optimizing the relationship between pixel intensity and distance from center. In particular, for a given center location, we find the Euclidean distance between each pixel and the center and fit a cubic smoothing spline with pixel intensity as the response variable and distance from center as the predictor variable. The optimization routine selects the center location for which the corresponding smoothing spline has a minimal sum of squared residuals. This step is based on the presumption that beads are radially symmetric and helps reduce variability in bead image metrics because the physical bead center, which conceptually exists in a continuous 2-d space, does not precisely correspond to any of the pixel locations, which exist in a discrete 2-d space.

Figure 1 presents this process for two cropped bead images. This illustration shows the effect of optimizing the bead center location on the fitted profile. In particular, the optimized profile (bottom right panel for each bead) shows a higher intensity at distances near 0, representing the brightness of the bead center, compared to the profile that treats the center of the cropped bead image as though it is the center of the bead. The bead in the left example illustrates the importance of modelling the bead center when the bead is far from the center of the cropped image, which can occur, for instance, if the bead is near the edge of the field of view. The bead in the right example illustrates the importance of even minor (i.e., sub-pixel) adjustments to the bead center location. Even though the two considered centers are only roughly half a pixel apart, the effect on the modelled center intensity, as seen in the difference between the red and blue profiles for the bead on the right, is roughly 20%.


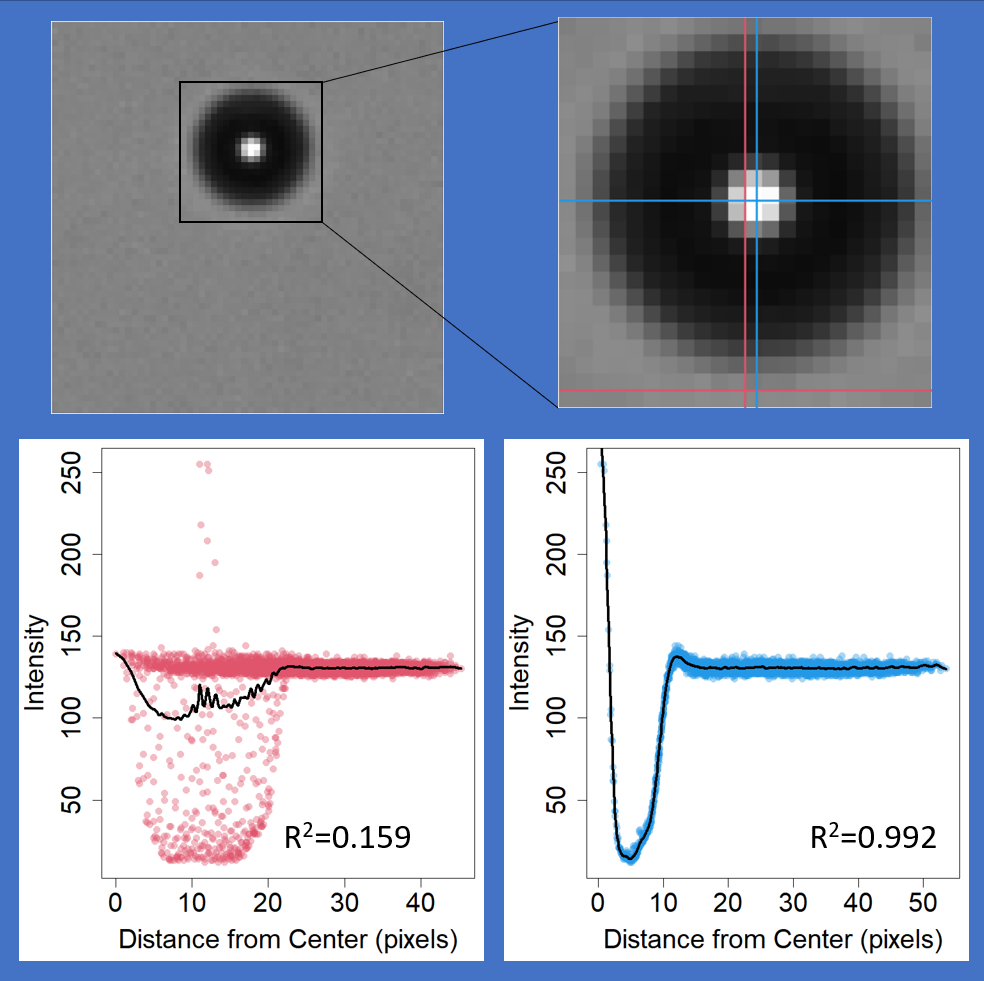

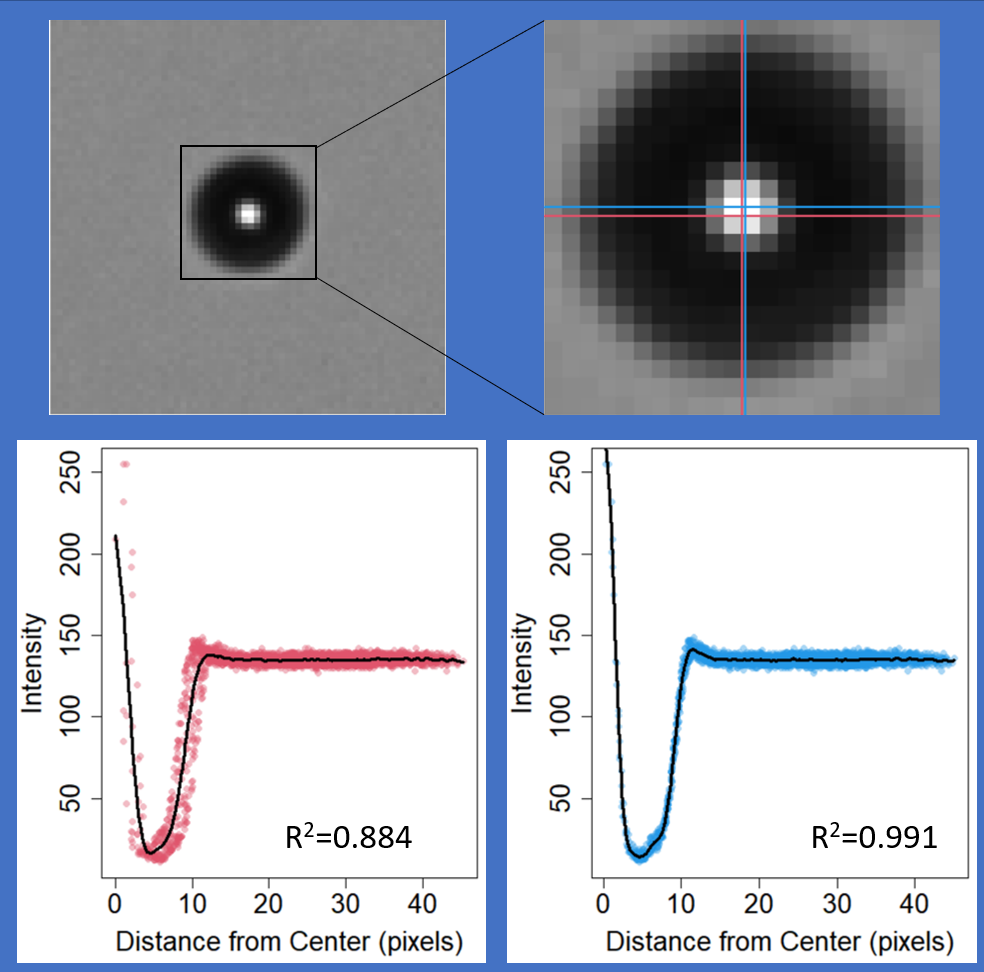


Supplementary Figure 1. (top left) 64 pixel x 64 pixel cropped bead image. (top right) Zoomed-in view of bead. Intersection of red lines depicts center of 64 x 64 image and intersection of blue lines depicts modelled center of bead. (bottom left) Relationship between pixel intensity and distance from image center. (bottom right) Relationship between pixel intensity and distance from modelled bead center.

Once profiles have been evaluated for all beads in a FOV image, we extract two features from the intensity profile for each bead, steepest slope and center intensity. The steepest slope feature is given by minimum value of the derivative of the smoothing spline. The center intensity feature is provided by the value of the smoothing spline at a distance of 1 pixel from the center. Each of these features are normalized by the average background to negate the effects of different exposure durations and spatial variability in the brightness across a given field of view. These aspects of the smoothing spline are illustrated in Figure 2.


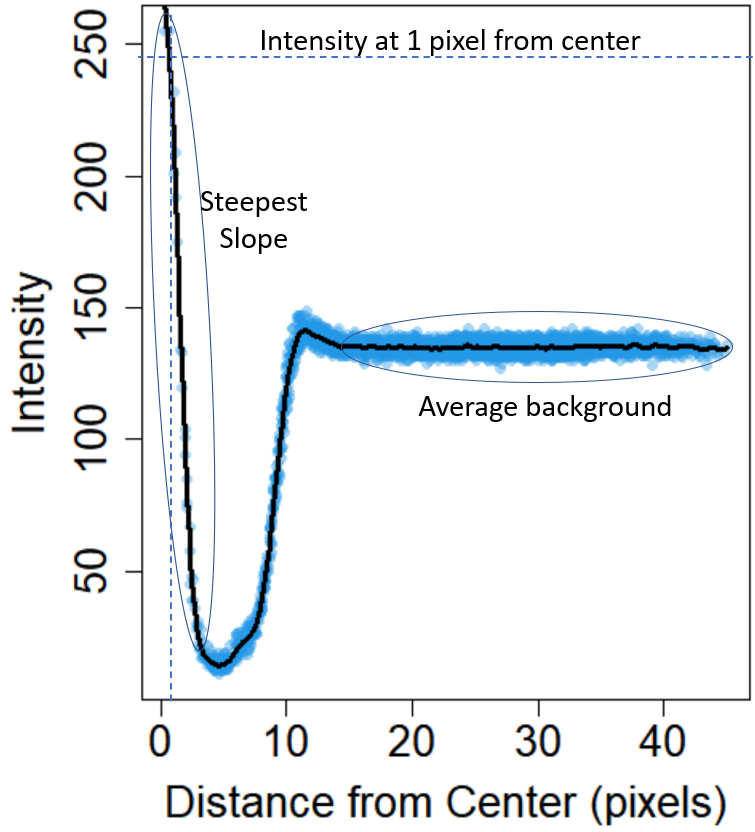


Supplementary Figure 2. Illustration of features extracted from bead intensity profile

For each of these features in turn, we perform a round of outlier removal within each combination of experiment, exposure, field of view, and Z, and take the median of the feature values among the remaining beads. We also record how many pixels are fully saturated in the median bead image(s), when ordered according to the considered feature. If the median image(s) contain no saturated values, we presume the reported median is unaffected by pixel saturation. We then evaluate the relationship between the median feature value and focal plane. We fit a cubic smoothing spline between these variables for the bead images in the first field of view from the A2K training set collected at exposure 12. Let this function be written as Y=f(Z), where Y represents median feature value, Z represents focal plane, and f() is the fitted cubic smoothing spline function. The process of IQR filtering, taking medians, and fitting a cubic smoothing spline is illustrated in Figure 3.


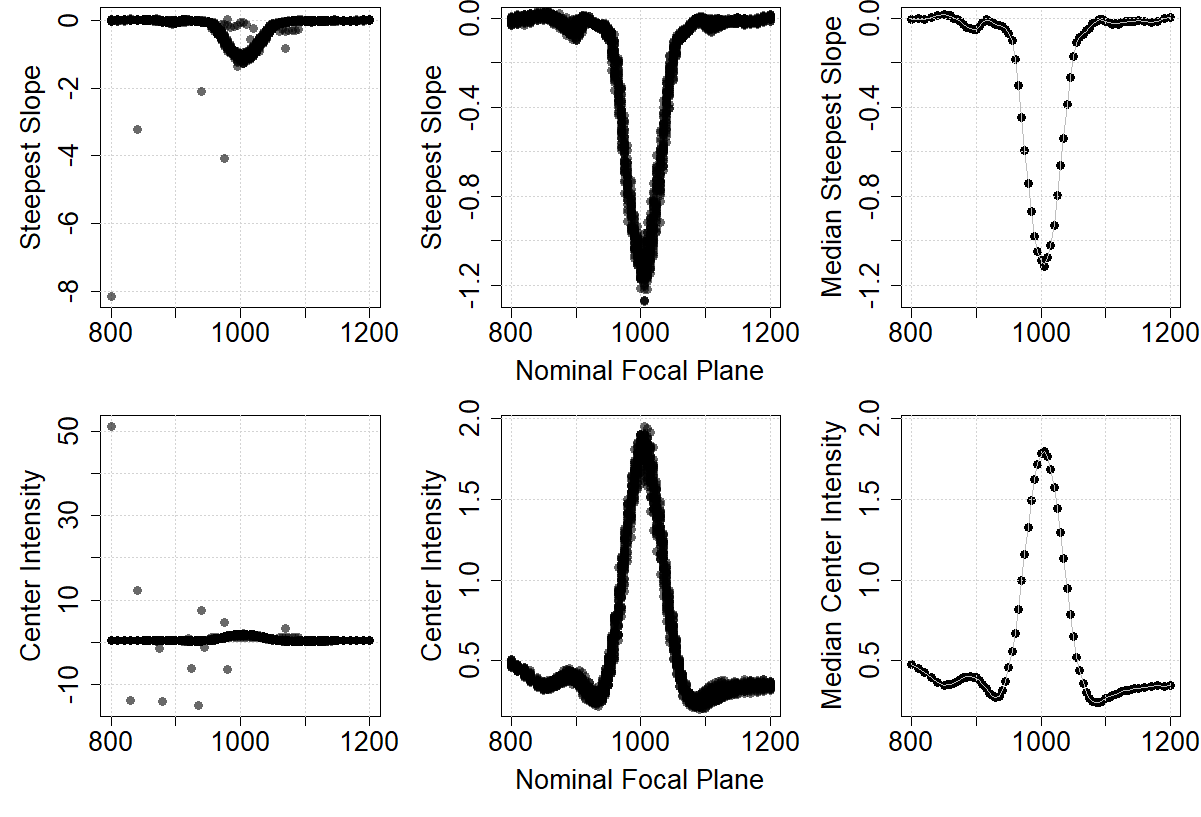


Supplementary Figure 3. Filtering and smoothing process for the steepest slope (top panels) and center intensity metrics (bottom panels). (left panels) Metric values from each bead in subset of the A2K training set collected from FOV 1 at an exposure setting of 12; (center panels) Metric values from subset of the A2K training set collected from FOV 1 at an exposure setting of 12 after applying IQR filtering; (right panels) Dots display median of IQR-filtered metrics data, taken at each distinct focal plane. Gray curves show cubic smoothing splines fit to displayed medians

We then consider data from each distinct combination of experiment, exposure, and field of view and use least squares to choose scalars s and d to minimize the sum of (s*Y-f(Z+d))^2, excluding (Y, Z) pairs where pixel saturation occurred. The resulting values of s and d are used to align the currently considered unsaturated data with the fitted profile from the first field of view for the A2K training set collected at exposure 12. That is, unsaturated median feature values (Y) and their corresponding focal plane values (Z) from the original dataset are mapped to s*Y and Z+d, respectively, to align with the data from the first field of view for the A2K training set collected at exposure 12. This process is illustrated for three combinations of experiment, exposure, and field of view in Figure 4.


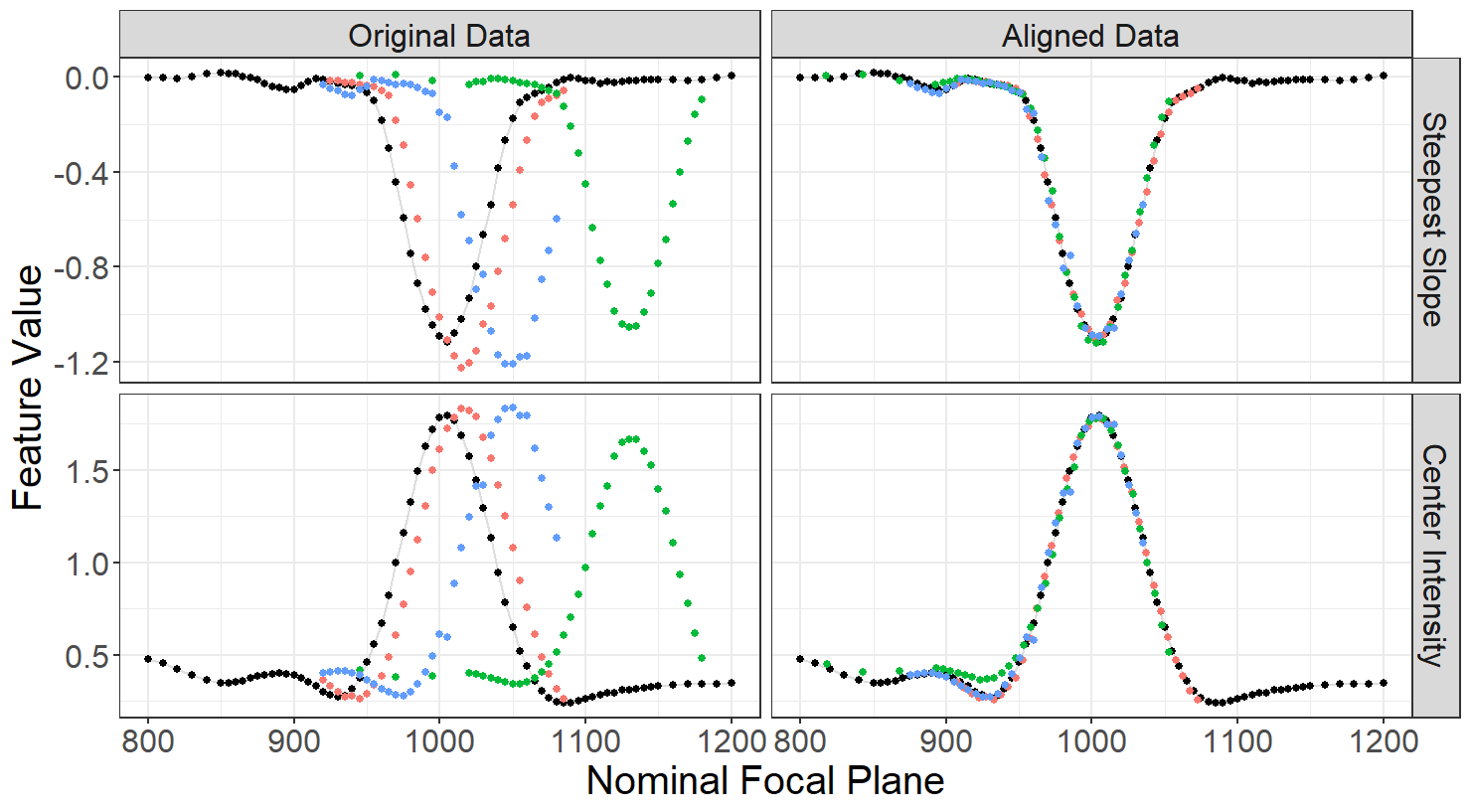


Supplementary Figure 4. Illustration of aligning profiles for the steepest slope (top panels) and center intensity metrics (bottom panels) across different combinations of of experiment, exposure, and field of view. Each of the four combinations of experiment, exposure, and field of view is given a different color. (left) Original metrics data, without aligning focal plane or rescaling metric values. (right) Aligned metrics data, where alignment to the black dataset is performed by allowing the focal plane values in the other datasets to be modified by an additive constant and allowing feature values in the other datasets to be modified by a multiplicative constant.

Once this has been completed for each field of view acquired on the currently considered microscope in turn, the combined, aligned data are used to fit a pooled profile, f_pool(), via a cubic smoothing spline, to describe the relationship between feature value and focal plane. The full set of aligned medians and the corresponding fitted cubic smoothing spline is illustrated in Figure 5.


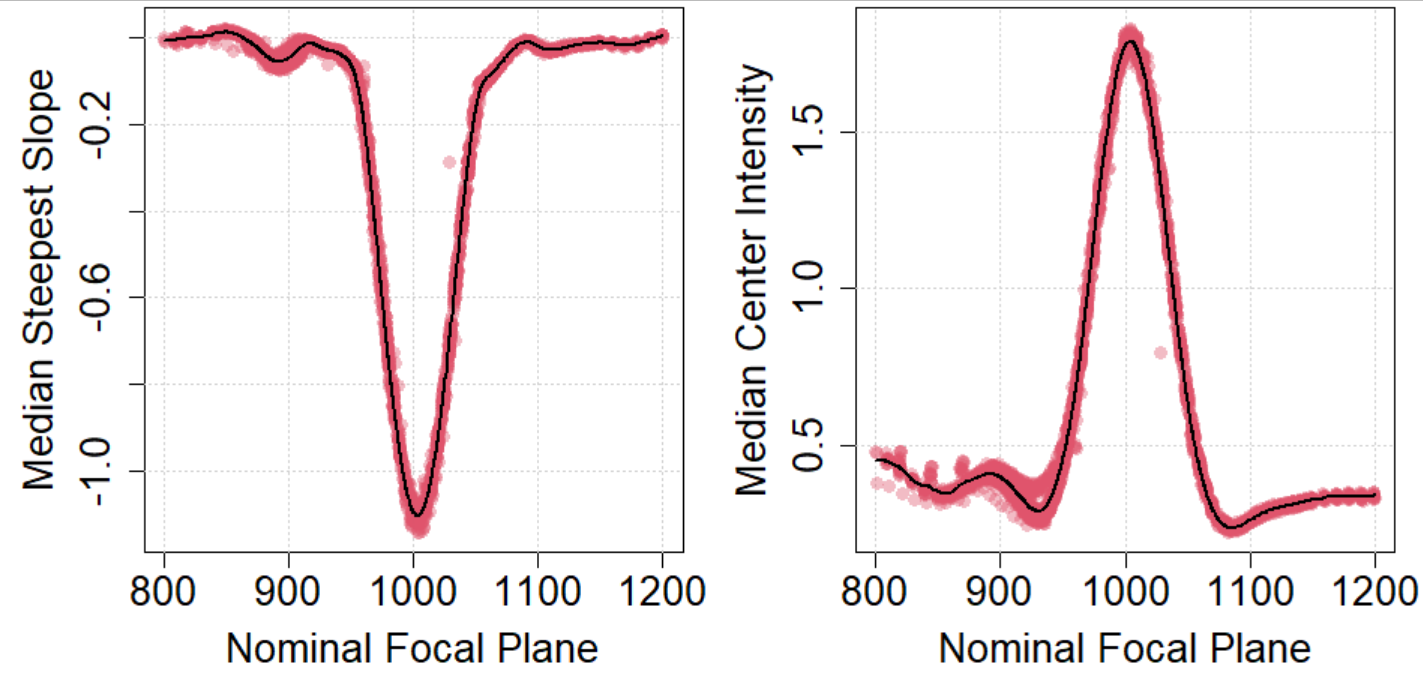


Supplementary Figure 5. Aligned median metrics data (omitting medians that correspond to images with saturated pixels) for the steepest slope (left) and center intensity (right) metrics. Red dots show the median values from each combination of experiment, exposure, field of view, and focal plane. Black curves show the fitted cubic smoothing splines, which provide the pooled profile for each feature.

For the steepest slope feature, denote the Z value corresponding to the minimum of the pooled profile as Z_pool. For center intensity, Z_pool is the Z value corresponding to the maximum of the pooled profile. Each field of view is considered in turn again to identify scalars s’ and d’ that minimize the sum (s’*Y-f_pool(Z+d’))^2, again excluding (Y, Z) pairs where pixel saturation may affect the median feature value. In the event that a dataset contained fewer than 6 distinct focal planes where saturation did not affect median feature values, we relaxed the constraint to the fewest number of saturated pixels that includes at least 6 distinct focal planes or as many focal planes as are included in the dataset, whichever is smaller. The feature-specific RFP was evaluated for each dataset as RFP_feature = Z_pool – d’. Plots showing the fit of the pooled profile to the median feature values for a few combinations of exposure, FOV, and experiment are shown in Figure 6.

The center panels of Figure 6 depict the benefit of using a pooled profile. For this experiment, images were collected at wider increments of focal plane and images collected near the RFP had saturated pixels, which can substantially impact the steepest slope and center intensity metrics. By using the pooled profile, we are still able to get a reasonable characterization of the RFP from the six focal planes for which saturation did not affect the median feature value. Additionally, the three datasets shown illustrate the stability of the shape of the relationship between focal plane and feature value, while the variability in peak locations across the three datasets shown illustrates that the nominal focal plane on the instrument is not an adequate indicator of the effective focal plane, even across experiments on a single microscope.

After completing this process using features of steepest slope and center intensity, respectively, a final RFP was defined for each combination of exposure, FOV region, and experiment as (RFP_max slope +RFP_center intensity)/2. These RFP values are the response variable used for training and testing the AI network.


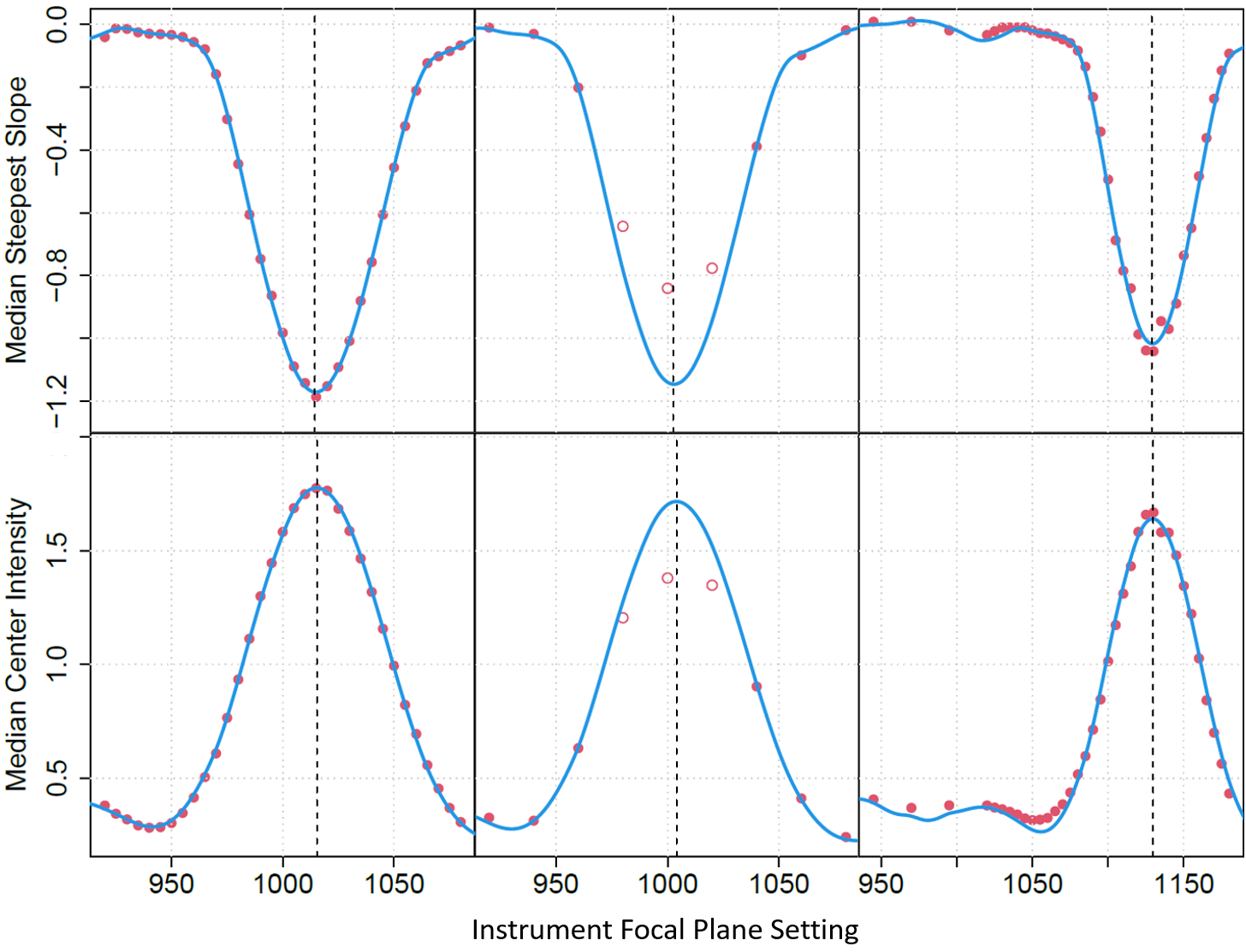


Supplementary Figure 6. Estimating the reference effective focal plane using two different metrics, steepest slope (top) and center intensity (bottom), in three different datasets: (left) Exposure 9 in FOV 4 from A2K test 1; (center) Exposure 9 in FOV 2 from A2K test 2; (right) Exposure 22 in FOV 3 from A2K test 4, which was acquired at a different lab. Red points depict median feature values. Hollow points indicate that at least one pixel was fully saturated in the median bead image(s) (sorted by feature value). Solid points indicate that no pixels were fully saturated in the median bead image(s) (sorted by feature value). Blue curves depict the pooled profile aligned to the solid points (i.e., the unsaturated medians). Vertical dashed lines depict the feature-specific reference effective focal plane for the displayed combination of exposure, FOV, and dataset.

## IQR Outlier removal

To achieve the highest possible accuracy from our regression network, the bead images taken at each Z and exposure level need to be very consistent. However, within the acquired images some beads do not sit properly at the bottom of the dish and thus are in a different focal plane than the rest of the beads. A few beads are attached to cells, lowering the gradient at the edges of those clusters. We compute the first metric as the maximum slope value between the center and the edge of the bead, the second metric is the average intensity around the optimized location of the bead center. The outlier removal of out of place beads is done using the interquartile range (IQR) applied to either metric 1 or 2. We eliminate any beads whose metrics values are more than 1.5 times the IQR above the third quartile or less than 1.5 times the IQR below the first quartile. IQR outlier removal is also applied to the ResNet18 predictions for individual bead images within each of the 10 ResNet 18 models. This method would filter about 5 % of data coming from an ideal normal distribution.
